# Modulation of biological motion perception in humans by gravity

**DOI:** 10.1038/s41467-022-30347-y

**Published:** 2022-05-19

**Authors:** Ying Wang, Xue Zhang, Chunhui Wang, Weifen Huang, Qian Xu, Dong Liu, Wen Zhou, Shanguang Chen, Yi Jiang

**Affiliations:** 1 State Key Laboratory of Brain and Cognitive Sciences, CAS Center for Excellence in Brain Science and Intelligence Technology, Institute of Psychology, Chinese Academy of Sciences, Beijing, China; 2 Department of Psychology, University of Chinese Academy of Sciences, Beijing, China; 3 Chinese Institute for Brain Research, Beijing, China; 4 Institute of Aviation Human Factors and Cognitive Neuroscience, Department of Aviation Psychology, Flight Technology college, Civil Aviation Flight University of China, Guanghan, China; 5 National Key Laboratory of Human Factors Engineering, China Astronaut Research and Training Center, Beijing, China; 6 China Manned Space Agency, Beijing, China; 7 Institute of Artificial Intelligence, Hefei Comprehensive National Science Center, Hefei, China

**Keywords:** Human behaviour, Perception

## Abstract

The human visual perceptual system is highly sensitive to biological motion (BM) but less sensitive to its inverted counterpart. This perceptual inversion effect may stem from our selective sensitivity to gravity-constrained life motion signals and confer an adaptive advantage to creatures living on Earth. However, to what extent and how such selective sensitivity is shaped by the Earth’s gravitational field is heretofore unexplored. Taking advantage of a spaceflight experiment and its ground-based analog via 6° head-down tilt bed rest (HDTBR), we show that prolonged microgravity/HDTBR reduces the inversion effect in BM perception. No such change occurs for face perception, highlighting the particular role of gravity in regulating kinematic motion analysis. Moreover, the reduced BM inversion effect is associated with attenuated orientation-dependent neural responses to BM rather than general motion cues and correlated with strengthened functional connectivity between cortical regions dedicated to visual BM processing (i.e., pSTS) and vestibular gravity estimation (i.e., insula). These findings suggest that the neural computation of gravity may act as an embodied constraint, presumably implemented through visuo-vestibular interaction, to sustain the human brain’s selective tuning to life motion signals.

## Abstract (web summary)

Utilizing spaceflight and its ground-based analog, the authors show how the Earth’s gravity sustains the human brain’s orientation-dependent sensitivity to biological motion signals based on neural computations of visual and vestibular gravitational cues.

## Introduction

As inhabitants of the Earth, humans have evolved under the constant influence of gravity. Without even noticing it, we keep our head aligned with the gravitational up and our feet pulled down towards the center of the globe. Indeed, almost everything we encounter or interact with is subject to the same gravitational constraint. This constraint, among the various environmental invariants, has infiltrated into our mental representation of the external world, formalizing psychological principles that mimic the physical laws to advance our cognitive, perceptual, and motor capabilities^1–4^.

Numerous studies have revealed a robust effect of representational gravity on visual memory, showing that the memorized position of a moving or an unsupported object was shifted downward, i.e., towards the direction of implied gravity^5^. Meanwhile, influences of the gravitational force were evident in the manual interception and time-to-collision estimation. In contrast to people’s deficient ability to intercept randomly accelerated objects or estimate arbitrary accelerations^6^, the timing of interceptive responses to free-falling balls was sufficiently precise and barely affected by visual deprivation^7–9^. Moreover, observers tended to trigger the catching movement earlier when the balls came from above instead of below, indicating that they applied a prior assumption that downward motion is accelerated by gravity^10^.

Intriguingly, the human brain’s selective tuning to gravity-constrained visual events also extends to the perception of biological motion (BM), the distinctive movement patterns initiated by living organisms that carry the signals of life. Human beings, like various other terrestrial animals, are endowed with the ability to readily extract messages transmitted by the BM signals, especially those from their conspecifics^11–17^. However, turning the stimulus upside-down severely impeded the detection and recognition of BM^18–24^, reduced the neural activity associated with BM representation^25^, and abolished the innate preference for BM in neonates and newly hatched chicks^26–28^, establishing the existence of a prominent inversion effect in visual BM processing. Remarkably, such inversion effect even occurred for an unusual body form orientation, e.g., waking on hands^29^, or for spatially scrambled BM stimuli devoid of any familiar shape^24,30^, suggesting that it may involve a brain mechanism devoted primarily to the analysis of kinematic cues. In particular, upright BM, especially the locomotion of articulated animals, consists of ballistic or pendular motion with acceleration profiles compatible with the effect of gravity. Thus, the inversion effect in visual BM perception is ascribed to a predisposed gravity bias in humans and other legged vertebrates, which may serve as a perceptual filter to enable efficient detection and appropriate interpretation of life motion signals^24,28^.

Arguably, the alleged gravity bias in life motion perception confers an adaptive advantage to all living creatures on Earth, including human beings. However, what gives rise to such perceptual bias, especially to what extent and how it is shaped by the Earth’s gravity field, remains largely unknown. It is possible that the inversion effect in BM perception, like that in face perception, is primarily built on our visual experience gained from long-term interaction with other tellurian animals throughout evolution. Alternatively, considering that our selective sensitivity to gravity-compatible BM signals has emerged and evolved in the Earth’s gravity environment, it is conceivable that this particular gravity environment (1 g) provides an indispensable, ongoing source for the BM inversion effect. On Earth, we achieve a continuous estimate of gravity’s orientation based on the real-time computation of vestibular and other bodily cues^31^. More particularly, the posterior part of the insular cortex, a core region responsible for vestibular processing, can respond to visually presented gravitational motion in an orientation-dependent manner. Such neural computation may provide a gravitational field vector along which gravitational acceleration could be identified during visual motion analysis, thereby acting as an online constraint to cultivate a selective sensitivity towards gravity-compatible BM patterns. If this is the case, we should expect to observe a stable BM inversion effect in 1 g gravity but a reduction of such inversion effect under reduced gravity conditions, mediated by a recalibration of the neural computation in the visual-vestibular network.

**Fig. 1:**
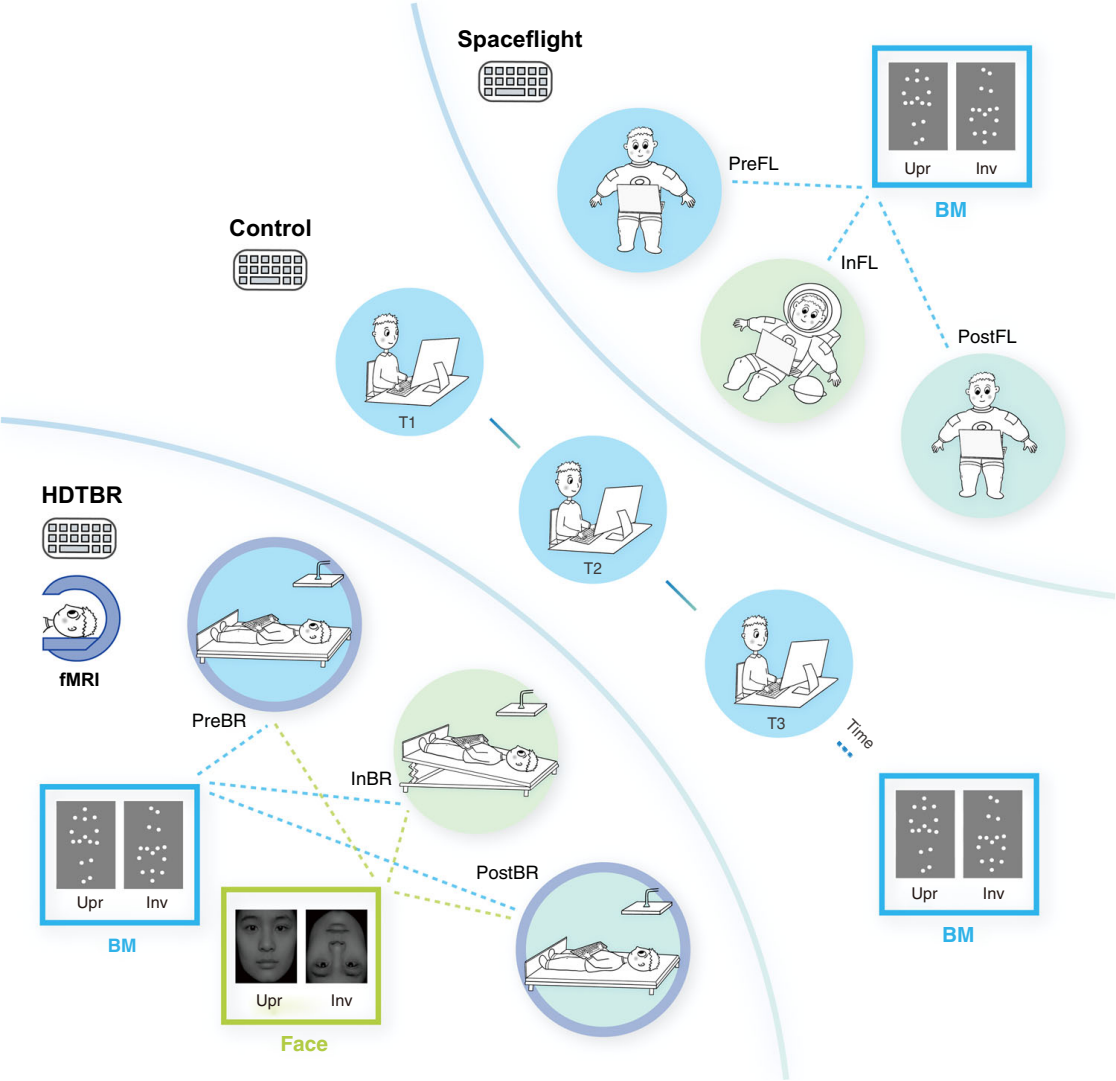
Schematic schedules of the space experiment (upper right panel), the ground-based control experiments (middle panel), and the 6° head-down tilt bed rest (HDTBR) space analog experiment (lower left panel). Participants performed a biological motion (BM) perception task before, during, and after the spaceflight/HDTBR, or throughout the control experiments. Besides, participants in the HDTBR experiment completed a face perception task in addition to the BM task during each test session and underwent two fMRI scanning sessions before and after the bed rest.

To examine these possibilities, we carried out a space experiment in which we assessed the BM inversion effect in astronauts exposed to microgravity during spaceflight (Fig. 1, Spaceflight). We also conducted ground-based control experiments in two groups of observers, one in an isolation environment (i.e., a simulated space capsule), one in a regular lab environment, to investigate the potential influences of non-gravity-related confounding factors such as test environment and practice effect (Fig. 1, Control). To support the behavioral results obtained from the space experiment and further investigate the neural responses in the human brain, we conducted a ground-based spaceflight analog experiment using the 6° head-down tilt bed rest (Fig. 1, HDTBR). Prolonged HDTBR can lead to the elimination of Gz gravitational stimuli on the body (i.e., head to toe G-stress) and the lack of work against the force of gravity by the bone, muscle, and cardiovascular systems in the vertical direction, which may cause changes in physiological responses^32,33^ and vestibular neural processing^34,35^ similar to what occurs with long-term spaceflight. It has been widely used by NASA and other international space agencies, in combination with the spaceflight recently^36^, to investigate the long-term influence of microgravity on humans.

If our superior sensitivity to upright relative to inverted BM is actively shaped by the neural computation of gravity, we predict that the BM inversion effect (i.e., the gravity bias) will decrease from pre- to in-flight in the astronaut group, get reduced with accumulating time in the HDTBR group, but yield no significant change in the control groups. We also assume that the neural activity associated with orientation-dependent BM representation will decrease after the HDTBR. Furthermore, if the vestibular gravity estimate is essential to the observed perceptual changes, we expect to observe altered functional connectivity in the visual-vestibular network underlying the perceptual changes.

## Results

### Space experiment

**Fig. 2:**
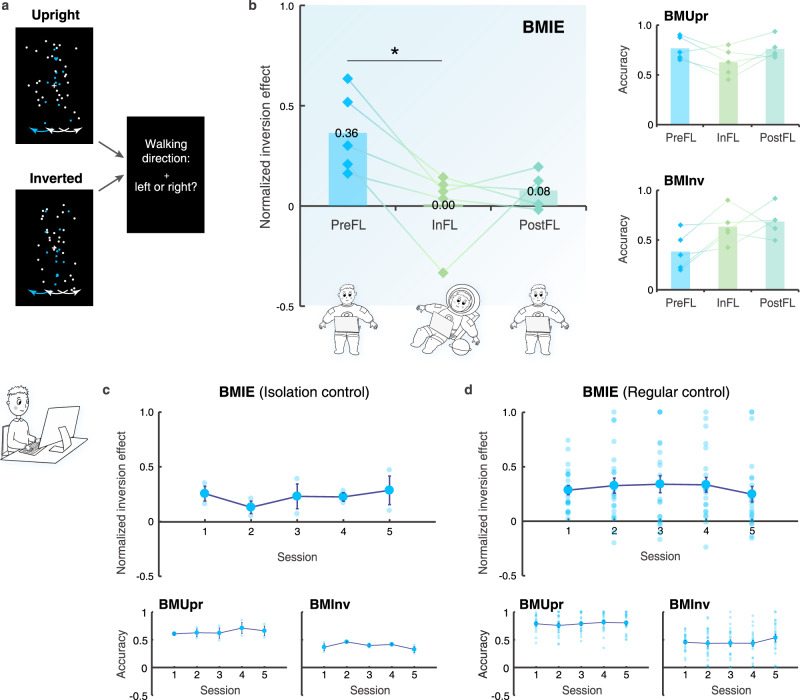
Experimental design and results for the space experiment and the ground-based control experiments. **a** Participants judged the walking direction of an upright or inverted point-light walker (rendered in blue for illustration only) embedded in a dynamic mask. The mask consisted of scrambled walkers with balanced left and right walking direction cues. Blue and white arrows represent the walking directions indicated by the target walker and the noise mask, respectively. Demos of sample BM stimuli are provided as supplementary information. **b** The normalized BM inversion effect (BMIE) and the task performance for the upright (BMUpr) and inverted (BMInv) conditions obtained before (PreFL), during (InFL), and following (PostFL) the spaceflight. Diamonds show individual data (*n* = 5). *p = 0.048 (two-tailed paired *t*-test, Bonferroni corrected). **c** Results obtained from the isolation control experiment where participants performed the BM perception task before (the first session), during (the second to the fourth session), and after (the fifth session) 30-day isolation. Blue dots represent individual data (*n* = 2). Error bars indicate ±1 SEM. **d** Results obtained from the regular control experiment where participants performed the BM perception task repeatedly in five test sessions over more than one month (35 days on average). Blue dots represent individual data (*n* = 22). Error bars indicate ±1 SEM. Source data are provided as a Source Data file.

The astronauts performed a BM perception task (Fig. 2a) before (PreFL), during (InFL), and after the spaceflight (PostFL). We divided the accuracy difference between the upright and inverted conditions by their sum to obtain a normalized BM inversion effect for each participant (Fig. 2b). A repeated measures ANOVA of the BM inversion effect revealed a significant main effect of the test phase (*F*(2, 8) = 8.39, *p* = 0.011). Remarkably, the inversion effect was largely diminished after about one week of spaceflight (InFL vs. PreFL: *p* = 0.048, adjusted for multiple comparisons by Bonferroni correction). Such impact was reduced but not eliminated half to one month after the astronauts returned to Earth (PostFL vs. PreFL: *p* = 0.073, Bonferroni corrected, also refer to supplementary results and Fig. S1 for more details). These results demonstrate a profound influence of microgravity exposure on the BM inversion effect, suggesting that the Earth’s gravity plays a pivotal role in sustaining the visual system’s orientation-dependent tuning to BM signals.

Further analysis revealed that spaceflight caused distinct change patterns in BM perception performances for the upright and inverted conditions (Fig. 2b). Overall, the test phase had a significant main effect on inverted BM perception (*F*(2, 8) = 5, *p* = 0.039) but less influenced upright BM perception (*F*(2, 8) = 2.74, *p* = 0.124). More specifically, for upright BM stimuli, the response accuracy declined slightly during spaceflight and returned to the normal level after the astronauts returned to Earth. By contrast, for inverted BM stimuli, the accuracy tended to increase during the flight and maintained at a relatively high level until half to one month after the flight (see also supplementary results and Fig. S1 for more details). The clear contrast between the two conditions suggests that microgravity intervenes with BM perception through an orientation-sensitive rather than a general adaptation mechanism.

### Control experiments

To further examine whether the test environment or practice effect could account for the observations from the space experiment, we administered the BM perception tasks to participants in two ground-based control experiments (Fig. 1, Control). For participants who were isolated in a simulated space capsule for 30 days, repeated tests caused no systematic change in the BM inversion effect or the separate performance on upright and inverted BM perception over time (Fig. 2c; Friedman’s Two-way Analysis of Variance on the inversion effect: χ^2^(4) = 2.8, *p* = 0.592; upright: χ^2^(4) = 3.6, *p* = 0.463; inverted: χ^2^(4) = 4.82, *p* = 0.306). We obtained similar results from another group of participants who performed the same task in a regular lab environment repeatedly over more than one month (Fig. 2d; repeated measures ANOVA on the inversion effect: *F*(4, 84) = 0.83, *p* = 0.512; upright: *F*(4, 84) = 0.76, *p* = 0.557; inverted: *F*(4, 84) = 1.55, *p* = 0.194). Results from these control experiments consistently suggest that non-gravity-related environmental factors or practice effects cannot account for the reduction of the inversion effect observed in the space microgravity environment.

### HDTBR experiment

**Fig. 3:**
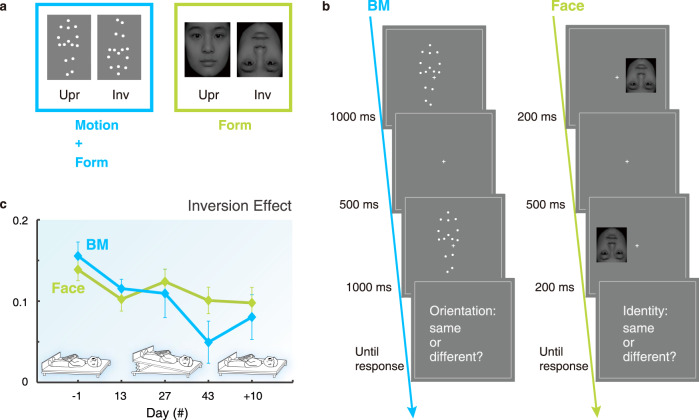
Experimental design and results for the behavioral tasks of the HDTBR experiment. **a** We measured the BM and face inversion effects, which were respectively related to both motion and form processing and form processing alone. **b** Procedures of the BM and face perception tasks in which participants indicated whether two successively presented walkers/faces were the same. **c** The change of the perceptual inversion effect along five test sessions before (day −1), during (day 13, 27, & 43), and after the bed rest (day + 10) for the BM (blue line, *n* = 12) and the face (green line, *n* = 15) perception tasks. Error bars indicate ±1 SEM. Source data are provided as a Source Data file.

To support the behavioral results obtained from the space experiment and further elucidate the neural mechanisms associated with the perceptual changes, we conducted a ground-based spaceflight analog experiment where a group of healthy participants completed a BM perception task (Fig. 3a, b) before, during, and after 45-day HDTBR. The behavioral results were highly consistent with the findings from the space experiment. The perceptual inversion effect changed significantly throughout the test sessions (Fig. 3c; *F*(4, 44) = 3.82, *p* = 0.009). There was an evident decrease of the inversion effect at the end of the HDTBR period (BR43 vs. BR-1: *p* = 0.038, Bonferroni corrected), which partially recovered 10 days after the bed rest (BR + 10 vs. BR-1: *p* = 0.284, Bonferroni corrected). Also, in agreement with the space experiment, the participants’ perceptual performance for the upright BM stimuli was rather stable across test sessions (*F*(4, 44) = 1.13, *p* = 0.353), while their performance for the inverted BM stimuli increased significantly due to the bed rest (*F*(4, 44) = 4.12, *p* = 0.006). Compared with the pre-bedrest baseline, the perception of inverted BM was marginally higher at the end of the bed rest period (BR43 vs. BR-1: *p* = 0.053, Bonferroni corrected), whereas such influence was no longer evident 10 days after the bed rest (BR + 10 vs. BR-1: *p* = 0.159, Bonferroni corrected).

To explore whether the current findings were specific to BM processing, we further compared the inversion effect in BM perception with the inversion effect in face perception (Fig. 3a, b). Faces, akin to BM, are of great biological significance and involve domain-specific neural mechanisms genetically wired in the human brain^37–40^. However, although robust face inversion effects occurred at both behavioral and neural levels^41,42^, such effects rely on the encoding of configural rather than kinematic information^43^. Therefore, if gravity specifically influences the visual sensitivity to gravity-constrained motion signals, we should expect a significant impact of HDTBR on the BM but not on the face inversion effect. In support of this assumption, prolonged HDTBR did not exert a reliable influence on the face inversion effect across the test sessions (Fig. 3c; *F*(4, 56) = 1.65, *p* = 0.174). Moreover, by looking into the pattern of change over time, we noticed that the face inversion effect did not show a decreasing trend from the second test session to the end of the HDTBR (linear trend test for the effect from BR13 to BR 43: *F*(1,14) = 0.01, *p* = 0.941), standing in stark contrast to the gradual change of the BM inversion effect during the same test period (linear trend test: *F*(1, 11) = 32.38, *p* < 0.001).

**Fig. 4:**
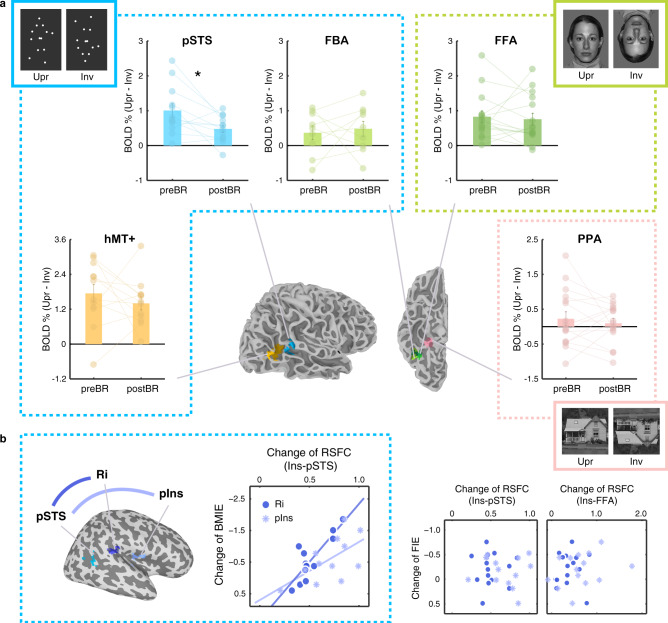
Results of ROI analysis based on the fMRI data obtained before and after the HDTBR. **a** The bar charts show the BM inversion effect in the pSTS, hMT + , and FBA, the face inversion effect in the FFA, and the house inversion effect in the PPA, averaged across participants. Colored dots represent individual data. Error bars indicate ±1 SEM. *: *p* = 0.019 (two-tailed paired t-test). A summary of the ROIs is displayed in a single participant. **b** The enhancements of resting-state functional connectivity (RSFC) between the pSTS and two insula ROIs (Ri: retroinsula; pIns: posterior insula) after vs. before the bed rest were significantly correlated with the change of the inversion effect in BM perception (BMIE). No reliable correlation was observed between such RSFC change and the change of the inversion effect in face perception (FIE), or between the Ins-FFA RSFC change and the change of perceptual FIE. Note that in a and b, we present data obtained from participants who showed valid performances in the corresponding behavioral task to facilitate the further correlation analysis (see methods for details; *n* = 12 in the BM condition for pSTS and MT + , *n* = 9 for FBA since no cluster could be identified in three participants due to noisy signals; *n* = 15 in the face condition for FFA, and *n* = 16 in the house condition for PPA). Additional results for ROI analysis based on data from all participants (*n* = 16) are shown in Fig. S1. Source data are provided as a Source Data file.

In addition to the behavioral measures, we acquired the participants’ neural responses to BM stimuli before and after the bed rest stage using functional magnetic resonance imaging (fMRI). As controls, participants also viewed face and house images during scanning. In line with the literature^25,42^, at the pre-bedrest stage, brain areas selectively tuned to BM and face signals showed higher responses to upright than inverted stimuli (Fig. 4a). Upright BM stimuli, relative to their inverted counterparts, activated the posterior superior temporal sulcus (pSTS) (*t*(11) = 4.95, *p* = 4×10^−4^); and upright faces, compared with the inverted ones, was associated with higher activation in the fusiform face area (FFA) (*t*(14) = 4.80, *p* = 3 × 10^−4^). For houses, there was no evident inversion effect (*t*(15) = 1.10, *p* = 0.29) in the parahippocampal place area (PPA)^44^. Crucially, after bed rest, the orientation-dependent responses to BM stimuli in the right pSTS decreased to a significant extent (*t*(11) = −2.75, *p* = 0.019), while no significant change was found for faces (*t*(14) = −0.38, *p* = 0.71) or houses (*t*(15) = −0.67, *p* = 0.516) in the corresponding cortical regions (Fig. 4a). These results, in accord with our behavioral findings, provide substantial evidence that HDTBR specifically reduces the gravity bias in visual BM processing.

Visual BM conveys kinematic and configural cues that are analyzed in distinct pathways converging at the pSTS^45,46^. It raises the question of whether neural activity in the motion-selective or body-form-selective areas could account for the decrease of the inversion effect observed at the pSTS. To address this issue, we extracted neural activity from the human motion complex (hMT+) and the fusiform body area (FBA)^47,48^, which respectively engage in the visual processing of body motion and body form information. The differences of neural signals in response to the upright and inverted BM were comparable before and after the bed rest in both the hMT+(*t*(11) = −0.88, *p* = 0.40) and the FBA (*t*(8) = 0.34, *p* = 0.739), indicating that the interaction between gravity and BM information analyses may not be completed before the stage where motion and form information are integrated into a coherent BM representation at the pSTS.

How does prolonged exposure to microgravity modulate the orientation specificity in visual BM representation? In the Earth’s environment, the human vestibular organs located in the inner ears monitor the linear and rotational accelerations of the head as well as the constant gravitational acceleration. The vestibular system further encodes head orientation relative to gravity and head motion and infers the position of the head in three-dimensional space, which contributes to a multitude of brain functions ranging from oculomotor and postural control, motion perception, spatial orientation, navigation to bodily self-consciousness^49,50^. The versatility of the vestibular system has been attributed to its multisensory nature. Unlike other sensory systems, there is no cortical area dedicated exclusively to the vestibular sense, and the vestibular inputs are integrated with the visual, motor, proprioceptive, and somatosensory signals throughout the thalamocortical vestibular pathways^49,51^. Within this multimodal network, the posterior part of the insular cortex is considered a core region since it can be activated by both vestibular stimulation and visually presented motion that carries gravitational acceleration cues^31^. This makes it a promising candidate to be responsible for the microgravity-induced recalibration of the gravity bias in visual BM representation. To test this possibility, we analyzed the resting-state functional connectivity (RSFC) between the pSTS and the insular cortex, which showed strengthened connectivity between these regions after prolonged HDTBR (Fig. 4b). Specifically, using the individual pSTS as a seed, we could identify two clusters in the right insula (retroinsula: Ri, voxel coordinates of peak activation: x = 41, y = −30, z = 21; posterior insula: pIns, voxel coordinates of peak activation: x = 38, y = −2, z = 16) that showed enhanced connection with the pSTS after the bed rest (*p* < 0.05 at the group level, uncorrected). These locations were coherent with the cortical regions critically involved in the computation of the gravity cue in 1 g motion^31^. More importantly, the elevated RSFC between the pSTS and these insula regions (defined as a Ri ROI and a pIns ROI for each participant) could well predict the decrement in the perceptual BM inversion effect (Fig. 4b; Ri: *r* = −0.84, *p* < 0.001; pIns: *r* = −0.61, *p* = 0.034), suggesting a key functional role of the insula-pSTS connectivity in the observed change of the gravity bias in BM perception. By contrast, there were no significant correlations between the enhancement of the pSTS-Insula connectivity and the change of the perceptual face inversion effect (Fig. 4b; Ri: *r* = −0.01, *p* = 0.972; pIns: *r* = 0.05, *p* = 0.878) and between the change of the face inversion effect and that of the FFA-Insula connectivity (Fig. 4b; Ri: *r* = −0.06, *p* = 0.825; pIns: *r* = −0.29, *p* = 0.322). Taken together, these results convergently suggest that the altered functional connectivity between cortical regions dedicated to visual BM representation and vestibular gravity estimation specifically underlies the attenuation of the gravity bias in BM perception.

## Discussion

Immersed in the planet’s gravitational field, humans are equipped with sophisticated cognitive capacities to cope with the effects of gravity. The rule that objects are attracted by the gravity of Earth has been so deeply ingrained that it distorts the visual representation of an object’s location^5^, facilitates the timing of free-falling motion^9,10^, and is thought to underlie the orientation specificity of visual BM perception^24,28^. Here we investigated how the gravity environment shapes our brain’s responses to visual BM signals, capitalizing on the manned spaceflight and HDTBR techniques. Specifically, we demonstrated that the BM perceptual inversion effect diminished after two weeks’ exposure to microgravity in space, and decreased gradually throughout a 45-day spaceflight analog using HDTBR. Moreover, we found an attenuated inversion effect in the neural representation of BM after the HDTBR. These findings provide substantial evidence that the Earth’s gravity facilitates the orientation-dependent visual perception of BM information.

In contrast to the significant change of the BM inversion effect, no such pattern emerged for the face inversion effect or the corresponding neural activity under simulated microgravity. These results are in line with the finding that the face inversion effect persisted in space despite impaired learning and recognition performances^52^. They are also consistent with the observation that the encoding of BM but not that of faces is affected by stimulus inversion relative to the gravitational frame of reference^53^. While the face inversion effect can be largely accounted for by compromised form processing^43,54^, the BM inversion effect involves a shape-independent component driven by the disruption of gravity-compatible motion invariants^24,55^. Hence, the reduced BM inversion effect following microgravity exposure probably results from the reduced gravitational influence on visual motion processing rather than form processing. In the 1 g environment, the internal representation of gravity may facilitate body kinematics analysis in an orientation-dependent manner. Prolonged microgravity exposure may reduce this influence and therefore diminish the BM inversion effect.

These findings link the physical effect of gravity to the mental constraints imposed on visual motion analysis, providing fresh insights into the origins of our abilities to process visual BM signals. The superior sensitivity to the movement of living creatures is an animal instinct^11–17^ that emerges from the first days or even the first hours of life^26–28,30^. Recent studies provide corroborative evidence for the existence of an innate mechanism for the visual analysis of BM by showing that individual differences in BM perception are under domain-specific genetic influences^37,38^. Here we further propose that the Earth’s gravity may play a vital part in the evolutionarily old mechanism underlying BM perception. More specifically, constant exposure to the planet’s gravitational field may exert selective pressure on its permanent inhabitants, thereby fostering the development of an ability to spot animated motions compatible with the effect of gravity.

The current study also extends our knowledge regarding the adaptive plasticity of the human perceptual system in the microgravity environment. Despite the solid evidence for potential health issues caused by microgravity exposure, far less clear is the impact of microgravity on perceptual and cognitive functions^56–58^. More specifically, there is little consensus about whether microgravity exposure would disturb the orientation-dependent effects in visual form perception^52,59–61^. The current study has identified a potent influence of microgravity on a specific aspect of visual information processing, i.e., BM perception, and indicated a mechanism by which the visual analysis of gravity-constrained kinematic rather than form cues adapts to the microgravity environment. Further studies are warranted to elucidate whether such mechanism is specific to BM processing or can be generalized to the visual analysis of non-biological gravitational motion. In addition, recent studies tracking the recovery trajectories of spaceflight-related neurocognitive changes reveal that most of these changes are (at least partially) reversible, albeit it may take weeks to months for some effects to recover to the baseline level^34,62^. Consistent with these results, our finding that the BM inversion effect did not fully recover several weeks after the spaceflight suggests that re-adaptation of brain functioning to the Earth’s gravitational field is a cumulative rather than an immediate process. The complete time course of such gravity-related neuroplasticity is a topic worthy of further research.

How did microgravity exposure modulate BM processing in the brain? The fMRI experiment showed a decrease of the inversion effect in the BM-selective region (i.e., the pSTS), but not in cortical areas responding to biological forms (i.e., faces and bodies) or general motion cues, suggesting that prolonged HDTBR specifically modulates the neural representation of BM information. Moreover, we found strengthened resting-state functional connectivity between the pSTS and the retroinsula and the posterior insula, with such altered visual-vestibular connectivity predicting the reduction of the perceptual inversion effect. Under the microgravity condition, the most prominent change in the sensory system is the lack of graviceptive stimulation, resulting in significantly different vestibular afferent signals relative to that under the normal gravity condition. It has been posited that the insular cortex in the vestibular system stores an internal model for analyzing visual gravitational motion based on graviceptive information^31^. This model represents a prior expectation about the effects of gravity, which could regulate online sensory information analysis (visual, vestibular, tactile, or proprioceptive) to assist in visual perception and manual interceptions^63^. In our experiments, prolonged HDTBR may induce adaptation of this internal model or modulate its implementation during visual BM analysis through the reweighting of sensory inputs^35^, thereby reducing the disparity between the visual responses to upright and inverted BM signals. Possibly, sensory reweighting that occurs in both the spaceflight and the HDTBR conditions leads to the similar perceptual outcomes observed in the current study^34,35^. However, since HDTBR is not identical to the spaceflight regarding the gravitational stimulation, future research on the spaceflight-induced changes of neural responses to BM stimuli in astronauts is needed to verify this assumption.

In sum, the current findings reveal that the human brain adaptively utilizes gravity as an embodied constraint to facilitate the perception of life motion signals. Throughout our evolutionary history, humans tend to incorporate the gravity force acting on the movements of other biological organisms and their own bodies into visual motion analysis. Such an embodied constraint can lead to superior perceptual processing of gravity-compatible kinematic cues and sustain the brain’s orientation-dependent tuning to life motion signals, conferring an evolutionary advantage to tellurian animals. Nevertheless, escaping from the Earth’s gravity may remove such a constraint through recalibrating the visual-vestibular connectivity, which provides an adaptive mechanism to help us better accommodate altered gravity environments.

## Methods

### Space experiment

#### Participants

Six astronauts (two females, mean age ± SD = 42 ± 6.9 years) participated in the space experiment. They were exposed to microgravity conditions for 13 or 15 days during the manned flight missions of China’s Shenzhou program. All but one (male) of these astronauts completed the tasks in all test sessions and were included in the data analysis. All participants gave informed consent in accordance with protocols approved by the Institutional Review Board of the China Astronaut Research and Training Center.

#### Stimuli and procedure

Participants were trained on the task prior to the experiment and tested before, during, and after the spaceflight. Table 1 showed the comprehensive test schedules for all participants. Each participant performed the task at least once and up to twice (in which case results were averaged) within each test session. Since there was no significant difference between the two post-flight sessions, we combined them into a PostFL condition in the main text and showed data from the post-flight1 and post-flight2 sessions in the supplementary results.

**Table 1:** The test schedules for the space experiment.

Subjects	Test sessions			
	Pre-flight	In-flight	Post-flight	
			Post-flight1	Post-flight2
S1	F−25	F5,F8	F + 2	F + 32
S2	F−25	F7,F10	F + 2	F + 32
S3	F−21,F−12	F8	F + 4	F + 12,F + 17
S4	F−21,F−12	F8	F + 4	F + 12,F + 17
S5	F−21,F−12	F5,F12	F + 4	F + 12,F + 17

*F* flight, numbers denote the No. of the test day relative to the spaceflight, ‘−’: pre-flight, ‘+’: post-flight.

Each test trial began with an intact point-light walker embedded in a scrambled mask presented at the center of the screen for 1 s. Participants were required to judge the locomotion direction of the target walker (left or right, counterbalanced across trials) by pressing one of two keys. The target walker was composed of 15 white dots located at the head and critical joints of a human figure walking on a treadmill without translational motion^64^. The scrambled mask was generated by randomizing the initial positions of the dots constituting the intact walker. Half of the dots in the mask were made of scrambled walkers in the same direction as the target walker, and the other half in the opposite direction, to eliminate the potential effects of directional cues conveyed by the mask (see^37^ Experiment 4 for details and supplementary files for demos of sample BM stimuli). There were 20 trials for each of the two experimental conditions, target upright and target inverted. For the upright condition, the number of dots in the mask was set to 30/75. For the inverted condition, the mask contained 15/30 dots. The noise level of the mask was counterbalanced across trials. The experiment programs were written and compiled in VB and VC.

#### Data analysis

We calculated a normalized perceptual BM inversion effect (BMIE) for each participant by dividing the accuracy (i.e., percent of correct responses) difference between the upright and inverted conditions by their sum. To reduce any potential interference, further analysis was only conducted with participants who exhibited a perceptual inversion effect (larger than 0) in the baseline/preflight session. The same rules applied to the analysis of all behavioral data obtained from the current study (including both the BM and face conditions) to facilitate comparisons across experiments and conditions. In the space experiment, all five participants showed a BMIE before the spaceflight and were subject to further analysis. Analysis of the behavioral data obtained from this study was performed by using SPSS20.

### Ground-based control experiments

#### Participants

Two healthy volunteers (2 males, mean age±SD = 35 ± 4.2 years) from the China Astronaut Research and Training Center participated in the 30-day isolation control experiment. Another twenty-four volunteers (12 females, mean age±SD = 22 ± 2.8 years) recruited among college students took part in the regular control experiment and got monetary payment for their participation. All participants had normal or corrected-to-normal vision and provided informed consent in accordance with protocols approved by the Institutional Review Board of the China Astronaut Research and Training Center (the isolation control experiment) and the Institutional Review Board of the Institute of Psychology, Chinese Academy of Sciences (the regular control experiment).

#### Stimuli and procedure

In two control experiments, participants performed the same BM perception task as in the space experiment but under the normal gravity (1 g) condition. In the isolation control experiment, participants executed tasks similar to what astronauts do for a space mission in a simulated space capsule on the ground for 30 days. Meanwhile, they performed the BM perception tests before, during, and after the isolation 14 times in total. The time intervals between any two successive tests were 2–6 days. To improve the reliability of the results and facilitate comparisons across experiments, data obtained from adjacent time points were averaged and combined into five test sessions: −10 to −1 day before the isolation (T1/baseline, with 2 tests), the 1^st^, 2^nd^, and 3^rd^ 10-day during the isolation (T2-T4, with 3 to 4 tests for each), and +1 to +10 day after the isolation (T5, with 2 tests). In the regular control experiment, participants performed the same task 6 times over more than one month (35 days on overage) in the lab. The intervals between any two successive test sessions were 6–9 days. Results from the first two tests were combined to achieve a stable baseline session (T1), followed by another four test sessions (T2-T4, with 1 test in each).

#### Data analysis

We calculated the BMIE for each participant in the same way as that in the space experiment. Also consistent with the space experiment, only participants who showed a stable inversion effect in the baseline session were subject to further analysis: 2 (in 2) for the isolation control experiment and 22 (in 24) for the regular control experiment.

### Ground-based HDTBR experiment

#### Participants

Sixteen healthy male volunteers (mean age ± SD = 26.6 ± 4.2 years) were recruited and paid for their participation in the HDTBR experiment. They underwent the 6° head-down tilt bed rest for 45 days, preceded by a 10-day adaptive phase to get familiar with the environment and tasks and complete the pre-bedrest test, followed by a 10-day recovery phase for the post-bedrest test. All participants had normal vision, with no family history of genetic diseases, and were free of neurological, psychiatric, or chronic health disorders. They provided informed consent in accordance with protocols approved by the Institutional Review Board of the China Astronaut Research and Training Center.

#### Stimuli and procedure

##### Behavioral assessments

Participants received training on the behavioral tasks prior to the tests. They were tested at five time points throughout the experiments, including one day before bed rest (BR-1), three different days during the bed rest (BR13, BR27, BR43), and ten days after bed rest (BR + 10). During the HDTBR period, participants lied on a specifically-made bed (rotated 6° relative to the horizontal plane) all the time to help them stick to the head-down tilt posture. They did all the daily activities and tests on the bed and were allowed to turn over to the prone position but never left the bed. At the pre- and post-bedrest stages, participants were free to move about but had to lie on the bed (with head and feet at the same height) when doing the perceptual tasks. The task procedure was kept consistent for all the test sessions.

Each test session consisted of four blocks to assess the perception of upright BM, inverted BM, upright face and inverted face stimuli, respectively. In the BM task, participants were required to judge whether or not two successively presented point-light walkers (1 s each, with a blank interval of an average of 500 ms) had the same walking direction by pressing one of two mouse buttons. The walkers were centrally located on the screen and subtended approximately 8.4 deg in height. The walking direction could be one of five angles: 10° left/right, 5° left/right, or 0° deviated from the vector pointing toward the viewer. Each direction was replicated 8 times for the walker displayed in the first interval, with a balanced number of same and different trials, resulting in a total of 40 trials within each block. The order of these trials was completely randomized. The procedure of the face perception task was similar to that of the BM task, except that a pair of same-gender face images were displayed (200 ms for each, to the left and right of the central fixation respectively) in each trial and participants had to indicate whether the faces had the same or different identities. The stimulus set consisted of 20 male and 20 female face images with the neutral expression, which were selected from the Chinese Affective Pictures System^65^. The gender (male/female) and location of the first face (left/right) were counterbalanced within each block. Visual stimuli were displayed on an LCD screen (1024 × 768 resolution; refresh rate: 60 Hz) hung above the head of the participants with a viewing distance of about 60 cm. Stimulus presentation and experimental manipulation were carried out using Matlab 2014b with the PsychToolbox-3 extensions^66^.

##### fMRI experiment

Participants underwent two fMRI scanning sessions, one prior to (BR-3) and the other following (BR + 3) the bed rest phase. The scanning protocol consisted of one resting state scan, two functional runs (with visual tasks) and an anatomical scan. During the task runs, upright and inverted visual stimuli from 3 categories, including BM, face, and house, were back-projected onto a screen inside the magnet bore. Participants viewed these stimuli via a mirror mounted on the head coil. The stimuli contained a set of point-light walkers^67^ facing toward 8 directions equally distributed between 0 and 180 degrees, 8 grayscale neutral face images from the NimStim face stimulus set^68^, and 8 house pictures. Each run was comprised of 18 blocks with 3 repetitions for the 6 conditions. The blocks were run in a pseudo-random order, interleaved with 2-s fixation intervals. Within each 10-s block, 10 exemplars selected randomly from the same category were displayed for 500 ms per item with 500-ms fixation intervals. Participants were required to perform a 1-back task within each block, i.e., press a button whenever the present stimulus was identical to the preceding one, to help maintain attention to the stimuli.

#### Behavioral data analysis

We calculated the BMIE and FIE for each participant in the same way as that in the space and the ground-based control experiments. Also consistent with those experiments, the analysis of behavioral data was based on results obtained from the participants who exhibited a perceptual inversion effect in the baseline (pre-bedrest) conditions, both for the BM perception task (*N* = 12) and the face perception task (*N* = 15). The same participants were included in the analysis of the fMRI data to calculate the correlation between the HDTBR-induced changes in behavioral performances and neural responses (see the result section in the main text). In addition, we also analyzed the fMRI data based on all 16 participants and presented the results in the supplementary file (see supplementary results and Fig. S2).

#### fMRI data acquisition and analysis

##### Data acquisition

The fMRI data were collected on a 3T Siemens Trio scanner equipped with a 12-channel phased-array head coil. High-resolution anatomical images were acquired using a 3D T1-weighted magnetization-prepared rapid-acquisition gradient echo (MPRAGE) sequence (1 × 1 × 1.33 mm^3^ resolution; 144 slices, no gap; TR/TE = 2530/3.39 ms, flip angle = 7°). Task and resting-state fMRI data were acquired using a 2D T2-weighted echo-planar imaging (EPI) sequence (3.125 × 3.125 mm^2^ in-plane resolution; image matrix = 64 × 64; 33 slices, 3.5 mm thickness, 0.7 mm gap; TR/TE = 2000/30 ms; flip angle = 90°).

##### Data preprocessing

Preprocessing and statistical analyses of fMRI data were performed using the Analysis of Functional NeuroImages (AFNI 17.0.11) package^69^ and MATLAB 2014b. The first two volumes of functional data from each run were discarded to allow for magnetization equilibrium. Spike noise in the signals was removed from the remaining volumes through interpolation. The functional data were corrected for slice timing and realigned to the volume acquired closest in time to the anatomical scan to correct for head movements. Low-frequency drifts were removed using a high-pass filter with a cut-off frequency of 1/128 Hz. The functional images and two structural images were registered to the average image of the two structural images and transformed into Talairach coordinate space using the TT_N27 template. The functional images were resampled to 3 × 3 × 3 mm^3^ resolution with a grey matter mask. The data were normalized with respect to the average signal of the entire run for each voxel, and modeled using multiple linear regression analysis for each condition and for each participant. Six motion parameters obtained from head motion correction were included as nuisance regressors.

##### ROI Analysis

The ROIs include the posterior superior temporal sulcus (pSTS), the fusiform face area (FFA), the parahippocampal place area (PPA), the fusiform body area (FBA), and the human motion complex (hMT+). We localized each of these ROIs for each participant based on the β values estimated for all conditions from all 4 functional runs, to avoid biases towards any test session or any orientation condition. The pSTS and FBA were identified with the contrast of (BM_upr+BM_inv) vs. (House_upr + House_inv); the FFA with (Face_upr + Face_inv) vs. (House_upr + House_inv); the PPA with (House_upr + House_inv) vs. (Face_upr + Face_inv); and the hMT+ with BM_inv vs. House_inv. Each ROI consisted of the most activated contiguous voxels (*q* < 0.05, FDR corrected, cluster size between about 135–675 mm^3^, i.e., 5–25 voxels) within the corresponding anatomical location reported in the literature^25,42,44,47^. Given the right lateralization of BM and face processing, only ROIs in the right hemisphere were considered for further analyses. No FBA cluster could be identified in three participants due to the large noise in the signals. Average peak MNI coordinates (x, y, z) for each ROI (valid participants only) were: pSTS (52, −44, 8), FFA (42, −44, −24), PPA (29, −33, −15), FBA (43, −39, −23), hMT + (51, −65, −1). For each participant, run, and condition, the raw time course of the fMRI signals was converted into a time course of percent signal change, relative to the average signal intensity for houses in the pSTS, FBA, hMT + , FFA, and that for faces in the PPA. Time courses of these BOLD signals were extracted from the most activated voxels of each ROI and corrected for baseline differences using the average signals of −2 and 0 s. BOLD responses from 4 to 10 s were averaged for the ROI analysis. The inversion effect was defined as the difference of neural activation between the upright and inverted conditions, following Kanwisher’s study on the face inversion effect^42^.

##### Resting-state functional connectivity

All preprocessing steps of resting-state functional data were consistent with those of the task-related fMRI data except for additional spatial smoothing with Gaussian kernel of 4 mm FWHM. The effects of head motion were removed through multiple linear regression. Voxel-wise mean and standard deviation of motion parameters (12 estimates) were included as nuisance regressors, and the residuals were considered clean resting-state fMRI signals. For resting-state data obtained before and after bed rest, the residuals were averaged across all voxels within each seed ROI. The functional connectivity between the seed and the whole brain was measured by Pearson’s correlation, with the r values transformed into Fisher’s Z scores. For each participant, a retroinsula (Ri) and a posterior insula (pIns) ROIs were localized respectively as a set of contiguous voxels within the posterior part of the right insula in TT_Daemon atlas showing enhanced connectivity with the pSTS after bed rest (*p* < 0.05, uncorrected, except that for one participant the pIns was located at a *p* value = 0.1). The average peak MNI coordinates (x, y, z) were: Ri (44, −28, 16); pIns (42, −3, 8). The change of functional connectivity strength after bed rest was calculated as the difference between the post-bedrest and pre-bedrest connectivity strengths divided by the sum of these values. Data within 3 standard deviations from the mean were included in further analysis to calculate the Pearson’s correlation coefficient between the change of functional connectivity and that of behavioral performances.

### Reporting summary

Further information on research design is available in the Nature Research Reporting Summary linked to this article.

## Supplementary information

Supplementary Information

Description of Additional Supplementary Files

Supplementary Movie 1

Supplementary Movie 2

Supplementary Movie 3

Supplementary Movie 4

Reporting Summary

## Data Availability

The data generated in this study have been deposited in the Institutional Knowledge Repository of the Institute of Psychology, Chinese Academy of Sciences (http://ir.psych.ac.cn/handle/311026/42020) and other relevant materials are available from the corresponding authors upon request. Individual data for astronauts were shown in Fig. 2. & Fig. S1 and not uploaded to an open source platform following the information security protocols of the China Astronaut Research and Training Center. Source data are provided with this paper.

## Source data

Source Data

## Code availability

The custom code used in the study for fMRI data processing with AFNI is publicly available via the Institutional Knowledge Repository of the Institute of Psychology, Chinese Academy of Sciences at: http://ir.psych.ac.cn/handle/311026/42020.
