# Supplementary material for: Modulation of biological motion perception in humans by gravity
Source: Nat Commun. 2022 May 19;13:2765. doi: 10.1038/s41467-022-30347-y (PMC9120521; doi:10.1038/s41467-022-30347-y)
Supplement: Supplementary file 1 — Supplementary Information [file 41467_2022_30347_MOESM1_ESM.pdf]

Supplementary Information for

Modulation of biological motion perception in humans by gravity

This file includes:

Supplementary results for the space and the HDTBR experiments

Supplementary Figures S1-S2

Legends for supplementary movies S1-S4

Supplementary results

Space experiment

Fig. S1 shows results for the BM perception task obtained in the four test sessions of the space experiment. The two post-flight sessions include tests conducted within 2-4 days and 12-32 days after the flight (For the complete test schedules, see Table 1). There was no significant difference between the two post-flight sessions in the BM inversion effect or in the performances for the upright and inverted conditions respectively (*ps* = 1, after Bonferroni correction for multiple comparisons).

To focus on the change induced by microgravity exposure, we further conducted a two-way repeated measures ANOVA with stimulus orientation (upright vs. inverted) and test phase (PreFL vs. InFL) as within-subject variables. Results showed that the interaction between orientation and test phase was significant ($F(1, 4) = 15.72$, $p = 0.017$), without any significant main effect (orientation: $F(1, 4) = 4.23$, $p = 0.109$; test phase: $F(1, 4) = 1.27$, $p = 0.323$). Post-hoc analyses revealed that microgravity significantly increased the perceptual performance in the inverted conditioned ($p = 0.036$) while slightly impaired the performance in the upright condition ($p = 0.071$).

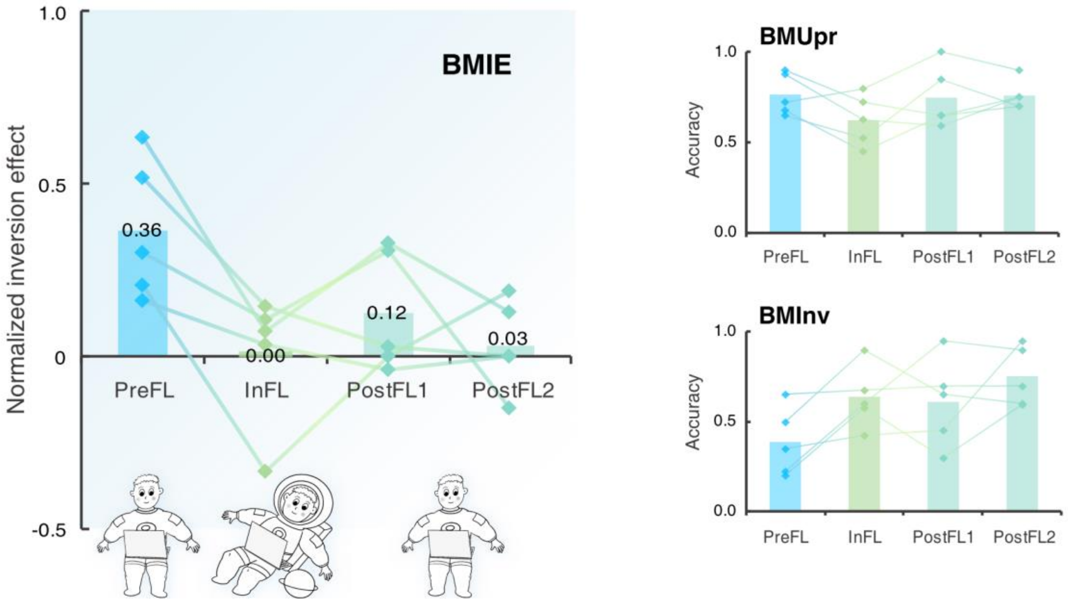

Fig. S1. Results of the space experiment in four test sessions. The left and right panels show the normalized BM inversion effect (BMIE) and the task performance for the upright (BMUpr) and inverted (BMInv) conditions obtained before (PreFL), during (InFL), and after (PostFL1 & PostFL2) the spaceflight. Diamonds represent individual data.

HDTBR experiment

Fig. S2 shows the ROI data obtained from 16 participants before and after the HDTBR. The FBA data includes 13 participants since no cluster could be identified in three participants due to noisy signals. Two-tailed t-test revealed that the difference of neural responses between the upright and inverted BM conditions (i.e., the inversion effect) in the STS decreased to a significant extent after bed rest ($t(15) = -2.15$, $p = 0.049$). By contrast, no significant change in the inversion effect was found for faces in the FFA ($t(15) = -0.26$, $p = 0.795$) and for houses in the PPA ($t(15) = -0.67$, $p = 0.516$). Moreover, the inversion effect in neural responses to BM stimuli did not changed after bed rest in the MT+ ($t(15) = -0.94$, $p = 0.362$) and the FBA ($t(12) = 0.65$, $p = 0.527$).

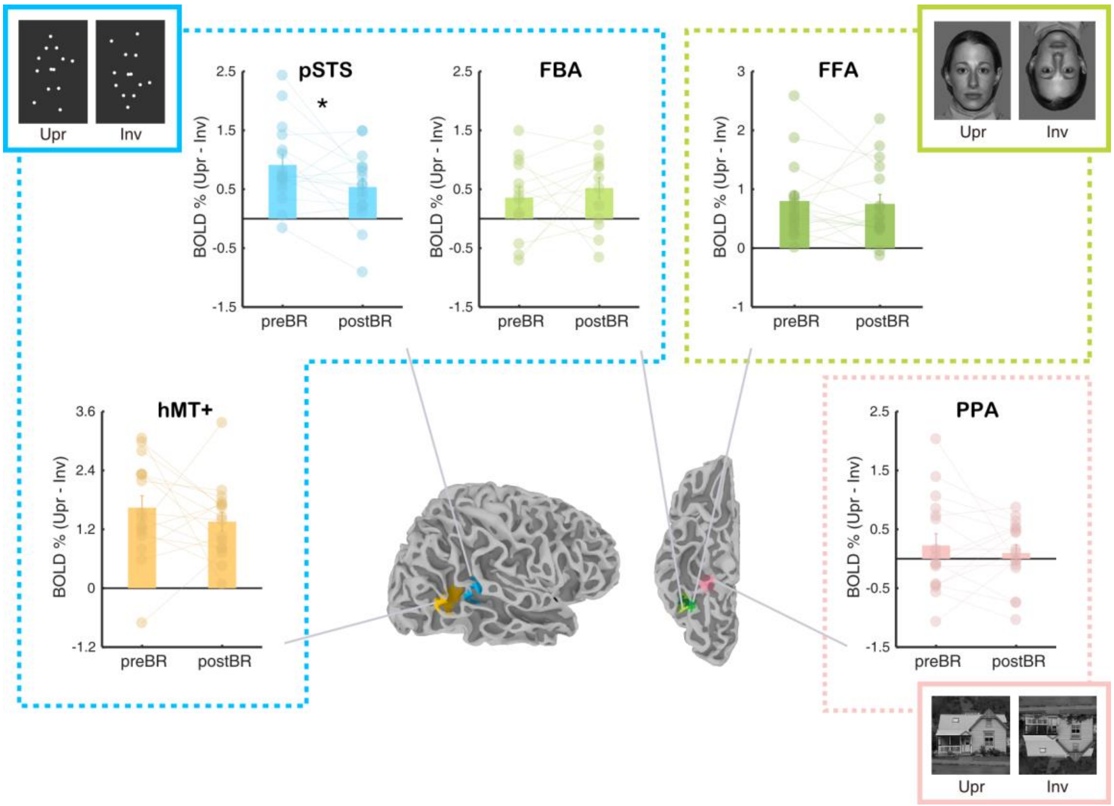

Fig. S2. Results of ROI analysis based on the fMRI data obtained before and after the HDTBR. a The bar charts show the BM inversion effect in the pSTS, hMT+, and FBA, the face inversion effect in the FFA, and the house inversion effect in the PPA, averaged across participants (n=16). Error bars indicate ±1 SEM. *: p < 0.05 (two-tailed paired t-test). A summary of the ROIs is displayed in a single participant. Source data are provided as a Source Data file.

Movie legends

Movie S1. A demo of upright BM stimulus masked by noise

Movie S2. A demo of inverted BM stimulus masked by noise

Movie S3. A demo of upright BM stimulus without noise

Movie S4. A demo of inverted BM stimulus without noise
