# Supplementary material for: Modulation of biological motion perception in humans by gravity
Source: Nat Commun. 2022 May 19;13:2765. doi: 10.1038/s41467-022-30347-y (PMC9120521; doi:10.1038/s41467-022-30347-y)
Supplement: Supplementary file 2 — Description of Additional Supplementary Files [file 41467_2022_30347_MOESM2_ESM.pdf]

Description of Additional Supplementary Files

File name: Supplementary Movie 1

Description: A demo of upright BM stimulus masked by noise

File name: Supplementary Movie 2

Description: A demo of inverted BM stimulus masked by noise

File name: Supplementary Movie 3

Description: A demo of upright BM stimulus without noise

File name: Supplementary Movie 4

Description: A demo of inverted BM stimulus without noise
