# Supplementary material for: Modulation of biological motion perception in humans by gravity
Source: Nat Commun. 2022 May 19;13:2765. doi: 10.1038/s41467-022-30347-y (PMC9120521; doi:10.1038/s41467-022-30347-y)
Supplement: Supplementary file 7 — Reporting Summary [file 41467_2022_30347_MOESM7_ESM.pdf]

nature portfolio

Corresponding author(s): Yi Jiang

Last updated by author(s): March 24, 2022

Reporting Summary

Nature Portfolio wishes to improve the reproducibility of the work that we publish. This form provides structure for consistency and transparency in reporting. For further information on Nature Portfolio policies, see our Editorial Policies and the Editorial Policy Checklist.

Statistics

For all statistical analyses, confirm that the following items are present in the figure legend, table legend, main text, or Methods section.

n/a	Confirmed	
☐	☒	The exact sample size (n) for each experimental group/condition, given as a discrete number and unit of measurement
☐	☒	A statement on whether measurements were taken from distinct samples or whether the same sample was measured repeatedly
☐	☒	The statistical test(s) used AND whether they are one- or two-sided<br>*Only common tests should be described solely by name; describe more complex techniques in the Methods section.*
☒	☐	A description of all covariates tested
☐	☒	A description of any assumptions or corrections, such as tests of normality and adjustment for multiple comparisons
☐	☒	A full description of the statistical parameters including central tendency (e.g. means) or other basic estimates (e.g. regression coefficient) AND variation (e.g. standard deviation) or associated estimates of uncertainty (e.g. confidence intervals)
☐	☒	For null hypothesis testing, the test statistic (e.g. F, t, r) with confidence intervals, effect sizes, degrees of freedom and P value noted<br>*Give P values as exact values whenever suitable.*
☒	☐	For Bayesian analysis, information on the choice of priors and Markov chain Monte Carlo settings
☒	☐	For hierarchical and complex designs, identification of the appropriate level for tests and full reporting of outcomes
☐	☒	Estimates of effect sizes (e.g. Cohen's d, Pearson's r), indicating how they were calculated

Our web collection on statistics for biologists contains articles on many of the points above.

Software and code

Policy information about availability of computer code

Data collection	MATLAB 2014b, Psychtoolbox-3
Data analysis	MATLAB 2014b, SPSS20, Analysis of Functional NeuroImages (AFNI 17.0.11)

For manuscripts utilizing custom algorithms or software that are central to the research but not yet described in published literature, software must be made available to editors and reviewers. We strongly encourage code deposition in a community repository (e.g. GitHub). See the Nature Portfolio guidelines for submitting code & software for further information.

Data

Policy information about availability of data

All manuscripts must include a data availability statement. This statement should provide the following information, where applicable:

- Accession codes, unique identifiers, or web links for publicly available datasets
- A description of any restrictions on data availability
- For clinical datasets or third party data, please ensure that the statement adheres to our policy

The data generated in this study have been deposited in the Institutional Knowledge Repository of the Institute of Psychology, Chinese Academy of Sciences (http://ir.psych.ac.cn/handle/311026/42020). Individual data for astronauts were shown in Fig. 2. & Fig. S1 and not uploaded to the open source platform following the information security protocols of the China Astronaut Research and Training Center. Source data are provided with this paper.

Field-specific reporting

Please select the one below that is the best fit for your research. If you are not sure, read the appropriate sections before making your selection.

☒ Life sciences ☐ Behavioural & social sciences ☐ Ecological, evolutionary & environmental sciences

For a reference copy of the document with all sections, see nature.com/documents/nr-reporting-summary-flat.pdf

Life sciences study design

All studies must disclose on these points even when the disclosure is negative.

Sample size	The sample size of the space experiment was comparable to that of previous studies that examined the microgravity-induced changes in perceptual performances in astronauts (e.g., de Schonen et al., 1998; Harris et al., 2017; Lipshits et al., 2000; N=3~7). The sample size of the regular-control experiment was about four times of that for the space experiment to obtain reliable results. The sample size of the HDTBR experiment was comparable to that of previous studies examining the HDTBR-induced changes in behavioral and neural responses (e.g., Cassady et al., 2016; Yuan et al., 2018; N=12~17). Note that sample sizes of the space experiment and the ground-based HDTBR experiment were not predetermined based on statistical methods, given the specific nature and limited availability of participants in these experiments.
Data exclusions	The current study aimed to test whether prolonged microgravity exposure or spaceflight analog would reduce the perceptual inversion effect typically found under the normal gravity condition. Therefore, for each experiment, only participants who exhibited a perceptual inversion effect in the baseline (pre-flight/control-baseline/pre-bedrest) conditions were subject to formal analysis. According to this pre-established criteria, we excluded no individual data from the space experiment, two from the regular-control experiment, four for the BM perception task and one for the face perception task from the HDTBR experiment. We have also confirmed that data analysis without excluding these participants did not change the patterns of the current results.
Replication	We did not replicate each experiment with the same manipulation in the current study. Whereas, we consistently found the reduction of BM inversion effect in the spaceflight and the HDTBR experiments and no systematic changes of this effects in the two control experiments.
Randomization	We used within-subject designs to examine how the inversion effect in BM perception changed with time under each experimental or baseline condition. Randomization is thus not applicable.
Blinding	Participants but not experimenters were blinded to the experimental design. Blinding is not supposed to be a relevant issue in the current study since all experiments were performed within subjects.

Reporting for specific materials, systems and methods

We require information from authors about some types of materials, experimental systems and methods used in many studies. Here, indicate whether each material, system or method listed is relevant to your study. If you are not sure if a list item applies to your research, read the appropriate section before selecting a response.

Materials & experimental systems

n/a	Involved in the study	
☒	☐	Antibodies
☒	☐	Eukaryotic cell lines
☒	☐	Palaeontology and archaeology
☒	☐	Animals and other organisms
☐	☒	Human research participants
☒	☐	Clinical data
☒	☐	Dual use research of concern

Methods

n/a	Involved in the study	
☒	☐	ChIP-seq
☒	☐	Flow cytometry
☐	☒	MRI-based neuroimaging

Human research participants

Policy information about studies involving human research participants

Population characteristics	Six astronauts (two females, mean age±SD = 42±6.9 years) who executed space missions participated in the space experiment, all but one (male) of them completed the tasks in all test sessions. Two healthy male participants (mean age±SD = 35±4.2 years) participated in the 30-day isolation experiment. Twenty-four (12 females, mean age±SD = 22±2.8 years) healthy participants were recruited and paid for their participation in the regular control experiment. Sixteen healthy male volunteers (mean age±SD = 26.6±4.2 years) were recruited and paid for their participation in the HDTBR experiment.
Recruitment	Participants of the space experiment were recruited among astronauts who executed a space mission. Participants of the isolation-control experiment were volunteers recruited from the China Astronaut Research and Training Center. Participants of the regular-control experiment were volunteers recruited among college students with monetary payment. Participants of the HDTBR experiment were paid volunteers recruited among normal, healthy populations.

Ethics oversight	All participants provided informed consent in accordance with study protocols approved by the Institutional Review Board of the China Astronaut Research and Training Center (the space experiment, the isolation-control experiment, and the HDTBR experiment) and the Institutional Review Board of the Institute of Psychology, Chinese Academy of Sciences (the regular control experiment).

Note that full information on the approval of the study protocol must also be provided in the manuscript.

Magnetic resonance imaging

Experimental design

Design type	block design for task-state fMRI; eye-closed resting-state fMRI
Design specifications	Participants underwent two fMRI scanning sessions, one prior to and the other following the HDTBR. The scanning protocol consisted of one resting-state scan, two task runs, and an anatomical scan. Each task run consisted of 18 blocks, with 3 repetitions for each of the 6 conditions (2 orientations: upright/inverted * 3 stimulus types: BM, face, house). These blocks were run in a pseudo-random order, 10-s for each, interleaved with 2-s fixation intervals.
Behavioral performance measures	Participants were required to perform a 1-back task within each block, i.e., press a button whenever the present stimulus was identical to the preceding one, to help maintain attention to the stimuli.

Acquisition

Imaging type(s)	functional and structural imaging
Field strength	3 Tesla
Sequence & imaging parameters	Task and resting-state fMRI data were acquired using a 2D T2-weighted echo-planar imaging (EPI) sequence (Imaging parameters: FOV = 200 mm × 200 mm, Matrix size = 64 × 64; Slice thickness: 3.5mm; TE=30ms; TR=2000ms; Flip Angle=90°).<br><br>High-resolution anatomical images were acquired using a 3D T1-weighted magnetization-prepared rapid-acquisition gradient echo (MPRAGE) sequence (Imaging parameters: 1 × 1 mm2 in-plane resolution; Slice thickness: 1.33 mm; TE=3.39ms; TR=2530ms; Flip Angle = 7°).
Area of acquisition	whole brain

Diffusion MRI ☐ Used ☒ Not used

Preprocessing

Preprocessing software	Preprocessing and statistical analyses of fMRI data were performed using the AFNI package. The first two volumes of functional data from each run were discarded to allow for magnetization equilibrium. Spike noise in the signals was removed from the remaining volumes through interpolation using 3dDespike. The functional data were corrected for slice timing and realigned to the volume acquired closest in time to the anatomical scan to correct for head movements using 3dVolreg.Then 3dFourier was used to remove low-frequency drifts using a high-pass filter with a cut-off frequency of 1/128 Hz. The functional images and two structural images were registered to the average image of the two structural images using align_epi_anat.py.<br>All preprocessing steps of resting-state functional data were consistent with those of the task-related fMRI data except for additional spatial smoothing with Gaussian kernel of 4mm FWHM using 3dMerge.
Normalization	The functional images were spatially aligned to Talairach standard space using @auto_tlrc in AFNI.
Normalization template	TT_N27 template,Talairach coordinate
Noise and artifact removal	For task-state data, six motion parameters obtained from head motion correction were included as nuisance regressors in multiple linear regression using 3dDeconvolve in AFNI.<br>For resting-state data, we followed the methods recommended by afni, putting the voxel-wise mean and standard deviation of motion parameters (12 estimates) into the multiple linear regression as nuisance regressors.
Volume censoring	Volume censoring was not performed on the data.

Statistical modeling & inference

Model type and settings	Univariate (linear regression)
Effect(s) tested	For task-state data analysis, we localized the pSTS, FFA, PPA, FBA, and hMT+ for each participant. For each participant, run, and condition, the raw time course of the fMRI signals was converted into a time course of percent signal change, relative to the average signal intensity for houses in the pSTS, FBA, hMT+, FFA, and that for faces in the PPA. Time courses of these BOLD signals were extracted from the most activated voxels of each ROI and baseline corrected using the average signals of -2 and 0 s. BOLD responses from 4 to 10 s were averaged for the ROI analysis. The inversion effect was defined as the difference of neural activation between the upright and the inverted conditions in each ROI.

For resting-state data, we calculated the change of connectivity strength between the pSTS and the retroinsula (Ri) and posterior insula (pIns) ROIs as the difference of the post-bedrest and pre-bedrest connectivity strengths divided by the sum of these values. Then we calculated the correlation between the change of resting-state functional connectivity and that of the behavioral inversion effect.

Specify type of analysis: ☐ Whole brain ☒ ROI-based ☐ Both

Anatomical location(s)

The ROIs were defined based on the β values estimated for all conditions from all 4 functional runs, to avoid biases towards any test session or any orientation condition. The pSTS and FBA were identified with the contrast of (BM_upr+BM_inv) vs. (House_upr + House_inv); the FFA with (Face_upr + Face_inv) vs. (House_upr + House_inv); the PPA with (House_upr + House_inv) vs. (Face_upr + Face_inv); and the hMT + with BM_inv vs. House_inv.

For each participant, a retroinsula (Ri) and a posterior insula (pIns) ROIs were localized respectively as a set of contiguous voxels within the posterior part of the right insula in TT_Daemon atlas showing enhanced connectivity with the pSTS after relative to before HDTBR.

Each ROI was constrained by an anatomical mask determined by automated labeling algorithms based on the TT_Daemon atlas.

Statistic type for inference (See Eklund et al. 2016)

general linear test, cluster-wise

Correction

FDR correction

Models & analysis

n/a	Involved in the study
☐	☒ Functional and/or effective connectivity
☒	☐ Graph analysis
☒	☐ Multivariate modeling or predictive analysis

Functional and/or effective connectivity

Pearson correlation and Fisher Z transformation
